# An Inquiry into the Nature and Causes of Lateral Deformity of the Spine; in Reference, More Especially, to the Pernicious Effects of Certain Moral and Physical Influences, Resulting from the Modern System of Female Education

**Published:** 1835-04-01

**Authors:** 


*An Inquiry into the Nature and Causes of Lateral Deformity of the Spine; in reference, more especially, to the Pernicious Effects of certain Moral and Physical Influences, resulting from the Modern System of Female Education.* By EDWARD W. DUFFIN, M.D. *Second Edition.*—London, 1835. 8vo. pp. 94.

THIS is a work, of which the first edition was reviewed a few years ago in the London Medical and Physical Journal. A clever writer once concluded a letter by apologising for its length, and declaring that he had not time to make it shorter: Dr. Duffin, profiting by this hint, has employed part of the intervening time in curtailing his book, and has produced the sensible and well-timed essay before us.

The absurdities systematically committed in the education of young females, as far as regards their physical development, may be conveniently discussed under three heads: Dress, Studies, and Amusements.

*Dress.* Dr. Duffin inveighs against tight stays, and homicidal lacing, but seems inclined to admit the use of well-made corsets, without back-bones or busks. He allows the former to be bad, but would let the minor evil pass for a good. To us it seems that the difference is but in degree, and the question is, whether the intercostals are to be wholly thrown out of work, or are to get a job now and then. This is certainly the head and front of the offences of the age in point of dress, and makes all other errors *in costume;* for, as the poet thought it natural that those who hated not

Bavius should admire the verses of Mævius, so it is reasonable that those who condemn one half of the human race to perpetual dyspnœa, should indulge in corn-creating shoes, and look with complacency on asphyxiating stocks.

*Studies.* On this point also our author is too lenient. He animadverts, with just severity, on the want of exercise at those schools where, as in the instance cited by Dr. Forbes, (p. 57-8,) the girls pass twelve or fourteen hours at their studies; but he does not remark, that these book-mills or music-factories make their victims silly as well as diseased. There is a sort of retributive justice in the fact, that those who would barter health for accomplishments are disappointed; and, like the sorcerers of the old romances, in their contracts with the enemy of mankind, though they pay the enormous price, they are balked of the trivial reward.

*Amusements.* Under this head Dr. Duffin has some judicious observations, which will please many of our readers even more than ourselves; for, in the matter of sports and games, we confess ourselves staunch conservatives. Our taste on these points is almost as antique as that of Martinus Scriblerus, who refused to sanction any game less than two thousand years old; and we should prefer the game of ball, as played by Nausicaë and her maids, in the Odyssey, to "the author's improvement on Dodd's ball exercise," as lithographed at page 65. The good old constitutional sports have a smack of youth and joyousness about them; while many of the modern substitutes have so constrained an air, that they seem the invention of some powdered and spectacled philosopher, and resemble a remedy rather than an amusement.

Our author says that, when children lie two in a bed, they should occasionally change sides, as lying always on the same side favours the production of crooked spine in the predisposed. How this may be, we know not; but we recollect that the same thing is asserted by Darwin, who, notwithstanding his airy theories, was certainly a diligent observer of nature.

We strongly recommend this treatise to those who are engaged in education; and would suggest that girls, when first going to school, instead of giving their instructress the customary silver spoon, should present her with a copy of this essay: it may be worth its weight in gold to her.

